# Supplementary material for: The Systems Biology Research Tool: evolvable open-source software
Source: BMC Syst Biol. 2008 Jun 29;2:55. doi: 10.1186/1752-0509-2-55 (PMC2446383; doi:10.1186/1752-0509-2-55)
Supplement: Additional file 1 — SBRT Archive. An archive of the current version of the Systems Biology Research Tool. [file 1752-0509-2-55-S1.zip › sbrt-1.4.0/doc/developers_guide/api/sbrt/shell/io/SerializedInputFile.html]

SerializedInputFile


|  |  |  |
| --- | --- | --- |
| **Overview** \| **Package** \| **Class** \| **Use** \| **Tree** \| **Deprecated** \| **Index** \| **Help** | |  |
| **PREV CLASS**   **NEXT CLASS** | **FRAMES**    **NO FRAMES**     **All Classes** |  |
| SUMMARY: NESTED \| FIELD \| CONSTR \| METHOD | DETAIL: FIELD \| CONSTR \| METHOD |  |


---


## sbrt.shell.io Class SerializedInputFile<E>

```
java.lang.Object
  sbrt.shell.io.SerializedInputFile<E>
```

**Type Parameters:**: `E` - the type contained in instances of this interface.

**All Implemented Interfaces:**: java.lang.Iterable<E>, InputFile<E>, SbrtFile

**Direct Known Subclasses:**: SerializedMapInputFile

---

``` public class SerializedInputFile<E> extends java.lang.Object implements InputFile<E> ```

This class is used to read input files containing serialized
objects.

**Author:**
:   This class was written and documented by
    Jeremiah Wright while in the Wagner lab.

---

**Constructor Summary**

`SerializedInputFile(java.lang.String fileName)`
  
          Constructs a new text input file using the provided argument.

`SerializedInputFile(java.lang.String fileName,
FileFormat fileFormat)`
  
          Constructs a new text input file using the provided arguments.


| **Method Summary** | |
| --- | --- |
| `void` | `close()`             Closes this file. |
| `protected  void` | `finalize()`             Disposes of system resources associated with this file when called by the garbage collector. |
| `java.lang.String` | `getCanonicalName()`             Returns the canonical name of this file. |
| `FileFormat` | `getFileFormat()`             Returns the format of this file. |
| `java.lang.String` | `getOriginalName()`             Returns the original name given to this file object. |
| `boolean` | `isClosed()`             Indicates if this file has already been closed. |
| `static boolean` | `isValidFormat(FileFormat fileFormat)`             Indicates if the provided file format is valid for a text input file. |
| `java.util.Iterator<E>` | `iterator()`             Returns an object iterator over this file. |
| `protected  SerializedFileIterator<?>` | `objectIterator()`             Returns an object iterator over this file. |

**Methods inherited from class java.lang.Object**

`clone, equals, getClass, hashCode, notify, notifyAll, toString, wait, wait, wait`

**Constructor Detail**

### SerializedInputFile

```
public SerializedInputFile(java.lang.String fileName)
```

:   Constructs a new text input file using the provided argument.

    **Parameters:**: `fileName` - the name of the file to be read.

---


### SerializedInputFile

```
public SerializedInputFile(java.lang.String fileName,
                           FileFormat fileFormat)
```

:   Constructs a new text input file using the provided arguments.

    **Parameters:**: `fileName` - the name of the file to be read.: `fileFormat` - the format of the file to be read.


**Method Detail**

### isValidFormat

```
public static boolean isValidFormat(FileFormat fileFormat)
```

:   Indicates if the provided file format is valid for
    a text input file.

    :   **Parameters:**: `fileFormat` - the file format to test. **Returns:**: `true` if the provided file format is valid for a text input file; `false` otherwise.

---


### getFileFormat

```
public FileFormat getFileFormat()
```

:   Returns the format of this file.

    :   **Returns:**: the format of this file.

---


### getOriginalName

```
public final java.lang.String getOriginalName()
```

:   Returns the original name given to this file object.

    :   **Specified by:**: `getOriginalName` in interface `SbrtFile`
    :   **Returns:**: the original name given to this file object.

---


### getCanonicalName

```
public java.lang.String getCanonicalName()
```

:   Returns the canonical name of this file.

    :   **Specified by:**: `getCanonicalName` in interface `SbrtFile`
    :   **Returns:**: the canonical name of this file. **See Also:**: `File.getCanonicalPath()`

---


### objectIterator

```
protected final SerializedFileIterator<?> objectIterator()
```

:   Returns an object iterator over this file.

    :   **Returns:**: an object iterator over this file.

---


### iterator

```
public java.util.Iterator<E> iterator()
```

:   Returns an object iterator over this file.

    :   **Specified by:**: `iterator` in interface `java.lang.Iterable<E>`
    :   **Returns:**: an object iterator over this file.

---


### close

```
public final void close()
```

:   Closes this file. If this file is already closed, then
    invoking this method has no effect.

    :   **Specified by:**: `close` in interface `SbrtFile`
    :   **Throws:**: `ApplicationException` - if an I/O error occurred while closing this file.

---


### isClosed

```
public boolean isClosed()
```

:   Indicates if this file has already been closed.

    :   **Specified by:**: `isClosed` in interface `SbrtFile`
    :   **Returns:**: `true` if this file is closed; `false` othewise.

---


### finalize

```
protected void finalize()
                 throws java.lang.Throwable
```

:   Disposes of system resources associated with this file
    when called by the garbage collector.

    :   **Overrides:**: `finalize` in class `java.lang.Object`
    :   **Throws:**: `java.lang.Throwable` - the Exception raised by this method. **See Also:**: `Object.finalize()`


---


|  |  |  |
| --- | --- | --- |
| **Overview** \| **Package** \| **Class** \| **Use** \| **Tree** \| **Deprecated** \| **Index** \| **Help** | |  |
| **PREV CLASS**   **NEXT CLASS** | **FRAMES**    **NO FRAMES**     **All Classes** |  |
| SUMMARY: NESTED \| FIELD \| CONSTR \| METHOD | DETAIL: FIELD \| CONSTR \| METHOD |  |


---
